# Structure Activity Relationship of Dendrimer Microbicides with Dual Action Antiviral Activity

**DOI:** 10.1371/journal.pone.0012309

**Published:** 2010-08-23

**Authors:** David Tyssen, Scott A. Henderson, Adam Johnson, Jasminka Sterjovski, Katie Moore, Jennifer La, Mark Zanin, Secondo Sonza, Peter Karellas, Michael P. Giannis, Guy Krippner, Steve Wesselingh, Tom McCarthy, Paul R. Gorry, Paul A. Ramsland, Richard Cone, Jeremy R. A. Paull, Gareth R. Lewis, Gilda Tachedjian

**Affiliations:** 1 Centres for Virology and Immunology, Burnet Institute, Melbourne, Victoria, Australia; 2 Starpharma Pty Ltd, Melbourne, Victoria, Australia; 3 Faculty of Nursing and Health Sciences, Monash University, Clayton, Victoria, Australia; 4 Faculty of Pharmacy and Pharmaceutical Sciences, Monash University, Parkville, Victoria, Australia; 5 Department of Microbiology, Monash University, Clayton, Victoria, Australia; 6 Department of Microbiology and Immunology, University of Melbourne, Parkville, Victoria, Australia; 7 Department of Medicine, Monash University, Melbourne, Victoria, Australia; 8 Department of Surgery (Austin Health), University of Melbourne, Heidelberg, Victoria, Australia; 9 Department of Immunology, Monash University, Melbourne, Victoria, Australia; 10 Department of Biophysics, Johns Hopkins University, Baltimore, Maryland, United States of America; University of Pittsburgh, United States of America

## Abstract

**Background:**

Topical microbicides, used by women to prevent the transmission of HIV and other sexually transmitted infections are urgently required. Dendrimers are highly branched nanoparticles being developed as microbicides. However, the anti-HIV and HSV structure-activity relationship of dendrimers comprising benzyhydryl amide cores and lysine branches, and a comprehensive analysis of their broad-spectrum anti-HIV activity and mechanism of action have not been published.

**Methods and Findings:**

Dendrimers with optimized activity against HIV-1 and HSV-2 were identified with respect to the number of lysine branches (generations) and surface groups. Antiviral activity was determined in cell culture assays. Time-of-addition assays were performed to determine dendrimer mechanism of action. In vivo toxicity and HSV-2 inhibitory activity were evaluated in the mouse HSV-2 susceptibility model. Surface groups imparting the most potent inhibitory activity against HIV-1 and HSV-2 were naphthalene disulfonic acid (DNAA) and 3,5-disulfobenzoic acid exhibiting the greatest anionic charge and hydrophobicity of the seven surface groups tested. Their anti-HIV-1 activity did not appreciably increase beyond a second-generation dendrimer while dendrimers larger than two generations were required for potent anti-HSV-2 activity. Second (SPL7115) and fourth generation (SPL7013) DNAA dendrimers demonstrated broad-spectrum anti-HIV activity. However, SPL7013 was more active against HSV and blocking HIV-1 envelope mediated cell-to-cell fusion. SPL7013 and SPL7115 inhibited viral entry with similar potency against CXCR4-(X4) and CCR5-using (R5) HIV-1 strains. SPL7013 was not toxic and provided at least 12 h protection against HSV-2 in the mouse vagina.

**Conclusions:**

Dendrimers can be engineered with optimized potency against HIV and HSV representing a unique platform for the controlled synthesis of chemically defined multivalent agents as viral entry inhibitors. SPL7013 is formulated as VivaGel® and is currently in clinical development to provide protection against HIV and HSV. SPL7013 could also be combined with other microbicides.

## Introduction

UNAIDS (2007) estimates that 33.2 million people are infected with human immunodeficiency virus (HIV) and half of these are women [1]. While male condoms and male circumcision have demonstrated efficacy in preventing HIV infection, negotiating the use of condoms can be difficult for women and circumcision does not directly prevent male to female HIV transmission [2], [3]. Accordingly, microbicides are being developed that prevent or reduce transmission of HIV and other sexually transmitted infections (STIs) when applied to the vagina or rectum [4]. Microbicide classes include nonspecific surfactants or detergents and acid buffering agents, moderately specific macromolecular anionic polymers that block HIV and other STIs, and HIV specific drugs that inhibit viral entry and reverse transcription [4]. We have been focused on the discovery and development of microbicides with broad-spectrum antiviral activity as there is a demonstrated correlation between herpes simplex type 2 (HSV-2) incidence and increased risk of HIV acquisition [5], [6]. Thus microbicides that block both HIV and HSV can potentially reduce HIV transmission either directly or indirectly by preventing HSV acquisition.

**Figure 1:**
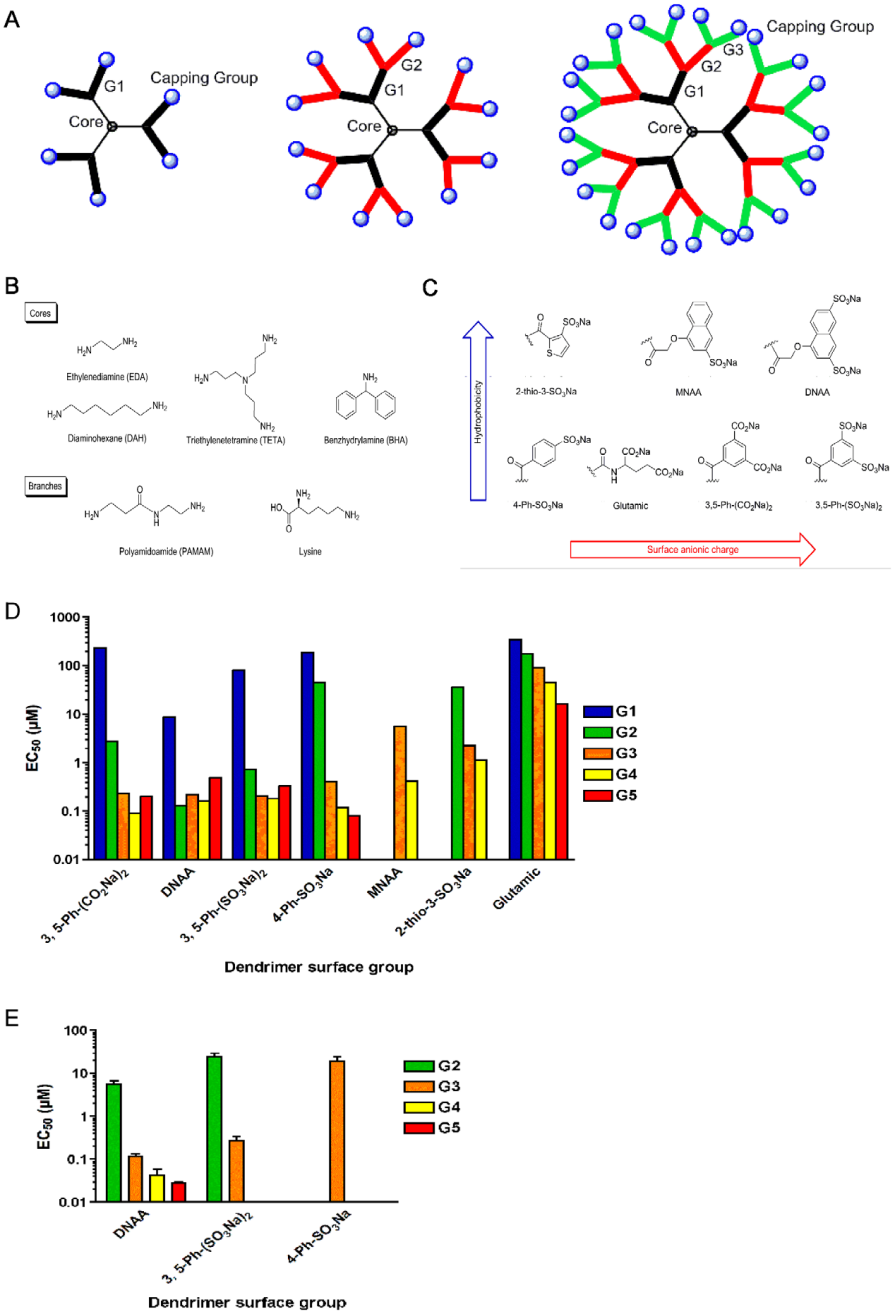
Dendrimer structure and SAR of L-lysine dendrimers against HIV-1 and HSV-2. (**A**). Model representation of dendrimer structure showing central core, branches in black, red and green for first (G1), second (G2) and third (G3) generations and surface groups denoted as blue round spheres. (**B**). Different types of cores and branches used in the synthesis of dendrimers. (**C**). Seven surface groups, L-glutamic acid, 2-thio-3-SO_3_Na, MNAA, 4-Ph-SO_3_Na, 3,5-Ph-(SO_3_Na)_2_, DNAA and 3,5-Ph-(CO_2_Na)_2_ evaluated in SAR studies. (**D**). SAR of L-lysine dendrimers against HIV-1 strain NL4.3 performed in MT-2 cells. G1–G5 dendrimers were evaluated for five of the seven surface groups except for MNAA where G1-G2 and G5 were not tested and 2-thio-3-SO_3_Na where G1 and G5 dendrimers were not tested. The EC_50_ values were obtained from at least two independent assays except for G1–G5 dendrimers with the glutamic surface group, G1 dendrimers with the 2-thio-3-SO_3_Na, 3,5-Ph(SO_3_Na)_2_, DNAA and 3,5-Ph-(CO_2_Na)_2_ surface groups and the G3 MNAA dendrimer, where one assay was performed. (**E**). SAR of L-lysine dendrimers against HSV-2 performed in HEL cells. G4-G5 and G2, G4-G5 were not tested for dendrimers with the 3,5-Ph-(SO_3_Na)_2_ and 4-Ph-SO_3_Na surface groups, respectively. EC_50_ values were obtained from at least three independent assays. Error bars denote standard error of the mean.

Recognised as a key building block of nanotechnology, dendrimers (dendri- = tree, -mer = branching) are a relatively new class of macromolecule characterised by highly branched, well-defined, three-dimensional structures that are being developed as drug delivery vehicles and as therapeutic agents [7], [8]. The controlled synthesis of dendrimers allows the assembly of highly defined, single molecule structures that radiate out in branches from a central initiator core (Figure 1A). The type of core and branching units (Figure 1B) can be altered to generate dendrimers of varying size and shape. In addition, dendrimer branches can be capped with different surface groups (Figure 1C) that can impart distinct biological and pharmacological properties. Thus dendrimers offer unique opportunities in the synthesis of agents with broad-spectrum antiviral activity [9]. Viruses rely on interactions with host receptors for binding and entry into target cells. Unlike small molecule drugs that tend to make monovalent contacts, dendrimers can bind to their target in a multivalent manner and overcome intrinsically weak monovalent interactions thus representing an attractive strategy for the development of viral entry inhibitors.

In this study we determined the structure-activity relationship (SAR) of dendrimers with regard to HIV-1 and HSV-2 inhibitory activity. We focused on dendrimers with L-lysine branches that have the dual advantage of greater stability and the capacity for synthesis as a pure species compared to previously reported approaches using polyamidoamine (PAMAM) linkages [10], [11]. Dendrimers were synthesized with 1 to 5 L-lysine branches (generations) and capped with one of seven different surface groups. The most potent dendrimers were selected for further evaluation in *in vitro* and *in vivo* studies and their mechanism of action was elucidated. Our SAR studies demonstrate that the dendrimer with the best overall profile with regard to potent dual action antiviral activity and potential to block cell-to-cell transmission of HIV was SPL7013, a fourth generation (G4) dendrimer with napththalene disulfonic acid (DNAA) surface groups. In contrast to linear polyanions, SPL7013 has similar potency against CCR5-(R5) and CXCR4-(X4) using strains of HIV-1. This dendrimer is the active pharmaceutical ingredient in VivaGel® currently in clinical development as a topical microbicide.

## Results

### Synthesis and purification of L-lysine dendrimers

Lysine dendrimers were prepared from a core comprising the benzhydryl amide of (L)-lysine using iterative cycles of Boc deprotection as described previously [12], [13]. All Boc-protected lysine dendrimers from generations one to five were chemically and physically stable and were prepared for subsequent surface functionalisation. This process involved Boc deprotection and formation of a carboxylic acid amide bond with one of seven possible surface capping groups: 4-sulfobenzoic acids sodium salt (4-Ph-SO_3_Na), L-glutamic acid (glutamic), 1,3,5-benzenetricarboxylic acid [3,5-Ph-(CO_2_Na)_2_], 3-sulfo-thiophene-2-carboxylic acid (2-thio-3-SO_3_Na), 3,5-disulfobenzoic acid sodium salt [3,5-Ph-(SO_3_Na)_2_], 2-[(3,6-disulfo-1-naphthalenyl)oxy]acetic acid disodium salt (DNAA) and 2-[(3-monosulfo-1-naphthalenyl)oxy]acetic acid sodium salt (MNAA). All dendrimers used in this study were >95% pure by HPLC and characterised by a variety of additional analytical techniques.

### HIV-1 inhibitory SAR studies with L-lysine dendrimers

The anti-HIV-1 SAR of L-lysine dendrimers was determined with respect to the number of generations and the surface groups (Figure 1C). Surface groups were examined with respect to anionic diversity (*i.e.* sulfonic acid or carboxylic acid) and lipophilic modifications (*i.e.* phenyl, naphthalene or alkyl). Dendrimers were evaluated for their capacity to inhibit replication of NL4.3, a HIV-1 strain that utilizes the chemokine coreceptor CXCR4 (X4 strain) for entry, in MT-2 cells. A positive control for HIV-1 inhibition was included in each assay to validate assay performance. The cytotoxicity of dendrimers was evaluated in the same assays.

The smallest dendrimers with potent anti-HIV-1 activity (defined as dendrimers with 50% effective concentration (EC_50_) values in the submicromolar range) were G2 dendrimers capped with DNAA or 3,5-Ph-(SO_3_Na)_2_, the former being more potent (Figure 1D and Table S1). In contrast, 3,5-Ph-(CO_2_Na)_2_ or 4-Ph-SO_3_Na surface groups conferred sub-micromolar inhibition of HIV-1 only when appended to G3 or larger dendrimers. Potent anti-HIV activity was observed for the MNAA surface group on a G4 dendrimer. The 2-thio-3-SO_3_Na and glutamic surface groups did not yield potent dendrimers. The selectivity index (SI) of the dendrimers was calculated to determine the specificity of HIV-1 inhibition at noncytotoxic concentrations (Figure S1). The G2 DNAA dendrimer had the greatest SI (SI = 940), while 3,5-Ph-(SO_3_Na)_2_ yielded the second most active G2 dendrimer (SI = 196) in MT-2 cells. These data demonstrate that dendrimers capped with a surface group imparting the greatest hydrophobicity and anionic charge density (i.e. DNAA, Figure 1C) were the most active particularly in the context of a smaller dendrimer. In addition, aryl sulfonic and carboxylic acid surface groups conferred the most potent HIV-1 inhibitory activity.

### HSV-2 inhibitory SAR studies with L-lysine dendrimers

We evaluated the HSV-2 inhibitory SAR of dendrimers focusing on surface groups that impart different degrees of hydrophobicity (*i.e.* phenyl vs naphthalene) and anionic density (*i.e.* one vs two sulfonic acid residues). HSV-2 inhibitory activity was determined in human embryonic lung (HEL) cells infected with the wild-type HSV-2 clinical isolate 250733 [14]. Dendrimer concentrations tested were not cytotoxic to HEL cells. Comparison of the HSV-2 inhibitory activity of G3 dendrimers capped with DNAA, 3,5-Ph-(SO_3_Na)_2_ and 4-Ph-SO_3_Na revealed that DNAA (EC_50_ ± standard error, 0.12±0.02 µM) and 3,5-Ph-(SO_3_Na)_2_ (0.26±0.08 µM, n = 3, p = 0.2) capped dendrimers demonstrated similar potency while dendrimers capped with 4-Ph-SO_3_Na (19.3±4.8 µM, n = 3, p = 0.05) was 160-fold less potent than DNAA capped dendrimers (Figure 1E). These data demonstrate that DNAA and 3,5-Ph-(SO_3_Na)_2_, the surface groups with the greatest hydrophobicity and negative charge density, were the most active against HSV-2.

Evaluation of G2–G5 dendrimers in the DNAA series revealed that anti-HSV-2 potency increased as dendrimers became larger, with the G5 dendrimer demonstrating the greatest potency (0.03±0.003 µM) (Figure 1E). This is in contrast to the anti-HIV-1 activity of the DNAA series where potency did not increase beyond G2 (Figure 1D). A similar relationship between increased dendrimer size and potency was also observed for inhibition of HSV type 1 (HSV-1, strain 250735) where the G4 DNAA dendrimer EC_50_ was 93-fold lower (0.13±0.03 µM, n = 10) than the G2 DNAA dendrimer (12.3±1.9 µM, n = 4, p<0.007). A similar pattern was also observed for the laboratory strains HSV-1(F) and HSV-2(G) (data not shown). Taken together these data demonstrate that there is a distinct difference in the HIV-1 and HSV-2 inhibitory SAR of dendrimers, and that they can be engineered with optimized potency for HIV-1 and HSV-2.

### Broad-spectrum anti-HIV activity of SPL7013 and SPL7115

**Figure 2:**
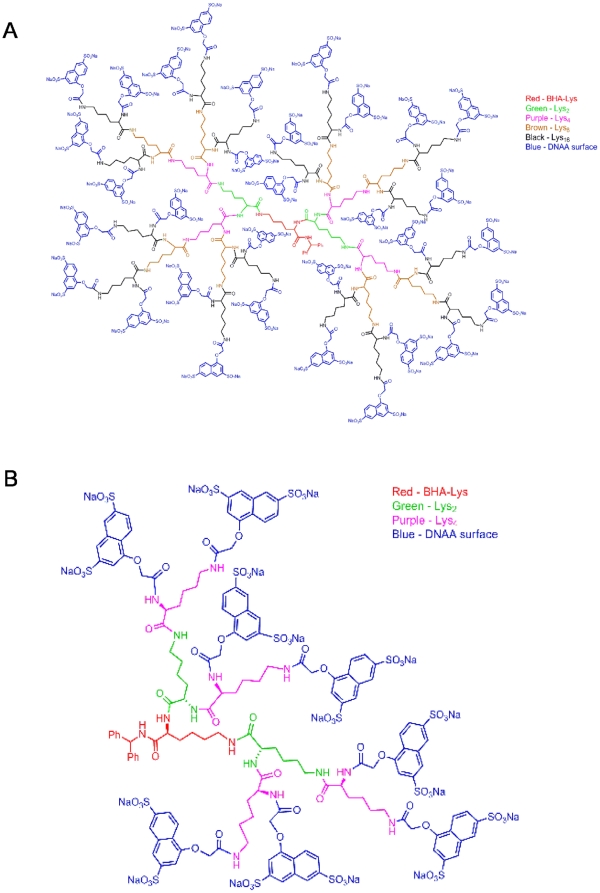
Chemical structure of SPL7013 and SPL7115. (**A**) Structure of G4 L-lysine dendrimer with DNAA surface groups and formula weight 16,582. (**B**) Structure of G2 L-lysine dendrimer with DNAA surface groups and formula weight 4,187.

HIV entry is initiated by binding of the gp120 surface glycoprotein to the CD4 receptor on the host cell which leads to conformational changes in gp120 and subsequent binding to the host cell CXCR4 or CCR5 chemokine coreceptors [15]. Interactions of gp120 with either CXCR4 or CCR5 drive conformational changes in the gp41 transmembrane glycoprotein resulting in fusion of viral and cellular membranes and viral entry. Sexual transmission of HIV is normally mediated by strains that utilize the CCR5 chemokine receptor [16]. Accordingly, we determined the inhibitory activity of dendrimers that were amongst the most potent inhibitors against NL4.3 in the SAR study (Figure 1D) for their inhibitory activity against the CCR5-using (R5) strain, HIV_Ba-L_, compared to NL4.3 in the TZM-bl cell line (Table S1). Of the six dendrimers tested, G2 (SPL7115) and G4 (SPL7013) dendrimers capped with DNAA were the most potent in inhibiting NL4.3 while SPL7013 was the most potent against HIV_Ba-L_ (Table S1). We selected SPL7013 (Figure 2A) and SPL7115 (Figure 2B) for further evaluation to compare the activity of a smaller G2 and a larger G4 dendrimer.

We investigated the ability of SPL7013 and SPL7115 to inhibit different HIV-1 clades circulating worldwide. Both dendrimers inhibited all HIV-1 clades (A, B, C, D, EA, F, G and O) and HIV-2 strains tested (Table 1 and Tables S2, S3 and S4). However, with the exception of NL4.3, where the activities of the dendrimers were similar, SPL7013 was 1.1–6.4-fold more potent in inhibiting HIV-1 compared to SPL7115 (Table 1) and demonstrated sub-micromolar inhibition for all strains tested (Table 1, Tables S2, S3 and S4). SPL7013 also showed similar inhibitory activity against both early circulating (NB25 and NB27) and late emerging (NB2 and NB6) R5 strains isolated from the peripheral blood mononuclear cells (PBMC) of HIV-1 infected individuals [17]. These data demonstrate that SPL7013 has broad-spectrum anti-HIV activity as demonstrated in both a cell line and in human PBMCs.

**Table 1:** SPL7013 and SPL7115 have broad-spectrum activity against HIV-1 strains and HIV-2 as determined in TZM-bl cells.

HIV Strain	Clade	Co-receptor usage^a^	Mean SPL7013 EC_50_ (µM)^b^	Mean SPL7115 EC_50_ (µM)^b^
NL4.3	B	X4	0.20±0.04	0.18±0.05
Ba-L	B	R5	0.26±0.05	1.08±0.28
92RW016	A	R5	0.14±0.06	0.65±0.31
92BR025	C	R5	0.07±0.01	0.45±0.07
92UG046	D	X4	0.22±0.08	0.99±0.49
CMU02	EA	X4	0.10±0.04	0.45±0.04
93BR020	F	Dual tropic	0.11±0.02	0.48±0.11
BCF01	O	R5	0.15±0.02	0.60±0.09
CB1-br	B	X4	0.05±0.02	0.27±0.07
MACS1-spln	B	Dual tropic	0.07±0.02	0.08±0.05
MACS3-LN	B	R5	0.08±0.02	0.45±0.12
MACS3-br	B	R5	0.09±0.03	0.51±0.19
NB2^c^	B	R5	0.19±0.05	0.66±0.10
NB6^c^	B	R5	0.09±0.01	0.32±0.02
NB25^f^	B	R5	0.10±0.01	0.53±0.16
NB27^f^	B	R5	0.10±0.01	0.47±0.02
HIV-2		X4	0.09±0.004	0.38±0.07

a X4 denotes HIV that uses the CXCR4 chemokine receptor for entry, R5 denotes HIV that uses the CCR5 chemokine receptor for entry and dual tropic use both X4 and R5 receptors for entry.

b 50% effective concentration (EC_50_) was determined in the TZM-bl indicator cell line from at least three independent assays.

c Late CCR5 HIV-1 virus isolated from PBMC of individuals with AIDS (CDC category IV disease) and CD4 counts <50 cells/µl.

f NB25 and NB27 are early CCR5 HIV-1 isolated from PBMC of individuals that was asymptomatic with CD4 counts >500 cells/µl (CDC category II disease) or was an acute seroconvertor with CD4 counts >750 cells/µl (CDC category I disease), respectively [17].

Linear polyanions are generally more potent at inhibiting X4 compared to R5 strains of HIV-1 due to interaction of the polyanion with the highly charged V3 loop on X4 strains [18], [19]. The lower potency of linear polyanions against R5 strains has been suggested as one explanation for their inability to prevent HIV acquisition in clinical trials [20], [21], [22]. Dendrimers are structurally and chemically distinct from linear polyanions, harboring a unique DNAA surface group. Accordingly, we determined whether SPL7013 and SPL7115 were more potent in inhibiting X4 compared to R5 HIV-1 strains (Table 1). We found no significant difference between the mean SPL7013 EC_50_ for X4 (0.14±0.04 µM, n = 4) and R5 (0.13±0.02 µM, n = 10, p = 0.07) strains. No significant difference was also observed for the mean SPL7115 EC_50_ values for X4 (0.47±0.18 µM, n = 4) and R5 (0.57±0.10 µM, n = 10, p = 0.14) strains. Since the presence of the cationic polymer DEAE-Dextran used to enhance HIV-1 infection in TZM-bl cells may differentially affect the dendrimer EC_50_ values for X4 and R5 strains [23], we also examined SPL7013 inhibition of HIV-1 from different clades in phytohemagglutinin (PHA) stimulated human PBMCs (Table S2). Similar to the TZM-bl data we observed no significant difference between the mean SPL7013 EC_50_ for X4 (0.12±0.03 µM, range 0.05–0.17, n = 4) and R5 (0.09±0.05 µM, range 0.02–0.15, n = 7, p = 0.18) strains. These data show that in contrast to polyanion polymers [18], [19], SPL7013 demonstrates similar potency in the inhibition of X4 and R5 strains of HIV-1.

We also evaluated the effects of human serum and human cervicovaginal secretions (CVS) on the HIV-1 inhibitory activity of SPL7013 in viral infectivity assays (Figure S2). In the presence of 40% human serum there was less than a 2-fold increase in the SPL7013 EC_50_ value compared to cultures infected in medium containing no human serum (Figure S2A). In addition, pooled human CVS had little impact on the SPL7013 EC_50_ with <3-fold increase compared to no CVS (Figure S2B). These data indicate that high protein or CVS is unlikely to have a major impact on the ability of SPL7013 to inhibit HIV-1 infection *in vivo*.

### Formulated SPL7013 (VivaGel®) demonstrates prolonged *in vivo* protection against HSV-2 in the mouse vaginal transmission model and does not cause toxicity that increases susceptibility to HSV-2

**Figure 3:**
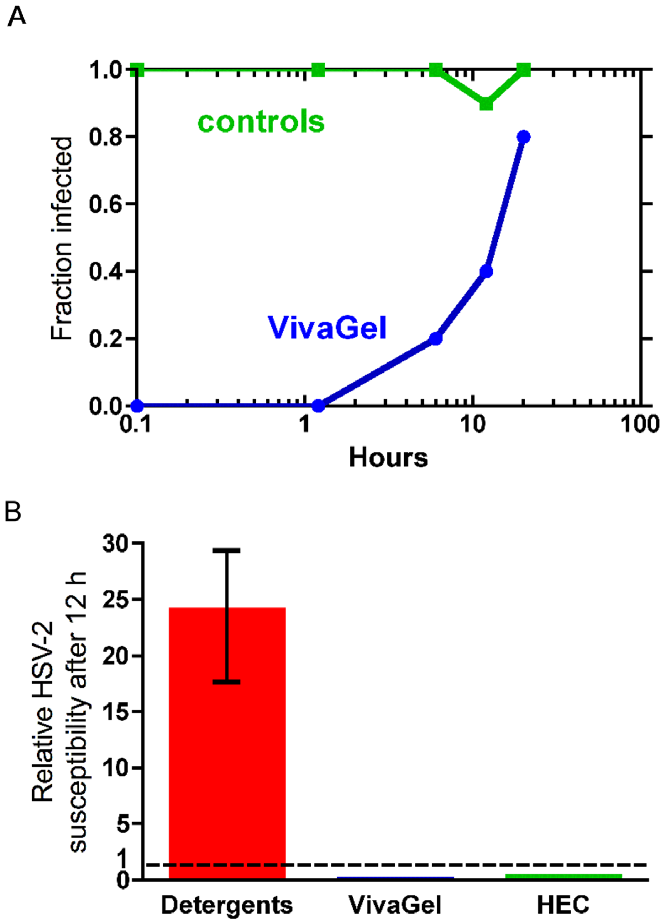
Formulated SPL7013, VivaGel®, protects against HSV-2 infection and does not increase susceptibility to HSV-2 in the mouse HSV-2 vaginal transmission model. (**A**) VivaGel® provides prolonged protection against HSV-2 infection. VivaGel® (or PBS control) was delivered vaginally to groups of 10 mice and the viral challenge (10 ID_50_) was delivered later at the times indicated. The viral inoculum infected 49 out of 50 control animals. (**B**) VivaGel® does not increase susceptibility to HSV-2. Groups of 40 mice were exposed to a single dose of VivaGel® and HEC placebo gel and after 12 h were challenged with a low dose (0.1 ID_50_) HSV-2 inoculum. As previously reported [25], animals exposed to nonoxynol-9 and several other candidate microbicide detergents become more susceptible to infection for many hours after exposure reaching a peak at about 12 h after a single exposure. The increase in susceptibility, as shown by the range bar, indicates the expected increase in vaginal transmission rate for a low-dose viral inoculum. In contrast 12 h after exposure to VivaGel® fewer animals were infected than in PBS control animals.

Previous studies have demonstrated that SPL7013 blocks HSV-2 infection in the mouse. However, these studies did not examine the longevity of the protection [24], which has implications with regard to how long before coitus the microbicide can be applied. Therefore we tested the ability of formulated SPL7013, VivaGel® (3% w/w SPL7013 in Carbopol gel at pH 4.5), to provide extended protection against vaginal transmission of HSV-2 in the mouse. VivaGel® provided prolonged protection lasting at least 12 h, and gave complete protection for more than 1 h (p = 0.01 at 12 h; p<0.001 at shorter times)(Figure 3A). These data are consistent with and extend a previous study demonstrating that formulated SPL7013 protects against vaginal HSV-2 infection when applied up to 60 min before viral challenge in the mouse [24].

Topical microbicides should not cause toxic effects that increase susceptibility to infections [16]. Mouse HSV-2 susceptibility models can detect any toxic effect, regardless of mechanism, that increases susceptibility to HSV-2 infection [25], [26]. In this model the animals are pretreated with Depo-Provera to expose living cells on the entire surface of the vagina making the mouse vagina more closely mimic the most accessible HSV-2 target cells in the human female genital tract. These are the columnar epithelial cells of the endocervical canal and regions of cervical ectopy that occur commonly in younger women, regions in which living cells are exposed directly on the face of the cervix. Mice exposed to a single dose of nonoxynol 9 and every other detergent-based candidate so far tested provide at most a few minutes of protection followed by several hours in which their susceptibility is greatly increased [25] (Figure 3B). In contrast, animals exposed to a single application of VivaGel® or the universal hydroxyethylcellulose (HEC) placebo gel did not demonstrate increased susceptibility to HSV-2 infection. These data are consistent with the lack of disruption of tight junctions between polarized epithelial cells by SPL7013 *in vitro* [27] and indicate that VivaGel® does not disrupt the mouse vaginal epithelium.

### Mechanism of HIV-1 inhibition by SPL7013 and SPL7115

**Figure 4:**
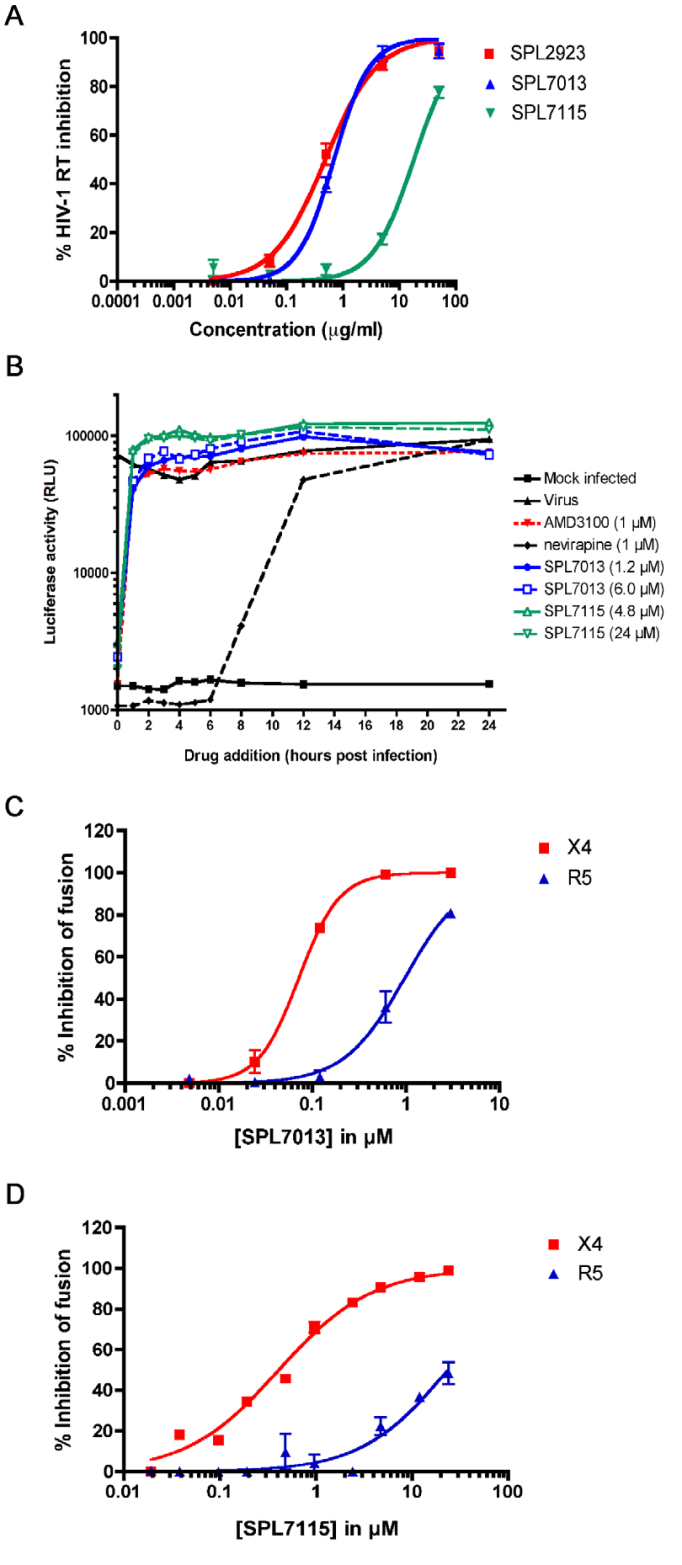
Mechanism of action studies of SPL7013 and SPL7115. (**A**) Inhibition of recombinant HIV-1 RT by dendrimers as determined using a heteropolymeric template-primer in the presence of 0.1 mg/ml BSA and 0.01% IGEPAL to prevent nonspecific binding of dendrimers to the RT. Data represent the mean of four independent assays. Error bars denote standard error of the mean. (**B**) Time of addition study performed in the TZM-bl indicator cell line with 20 and 100 µg/ml of SPL7115 and SPL7013. AMD3100 and nevirapine were included as controls for an entry and HIV-1 RT inhibitor, respectively. Data is a representative assay from one of three independent assays. (**C**) Inhibition of R5 and X4 gp120 mediated cell-to-cell fusion by SPL7013 and (**D**) SPL7115. Data from fusion assays are the average from at least three independent assays. Error bars denote standard error of the mean.

While SPL7013 and SPL7115 are anticipated to inhibit HIV-1 infection by preventing viral entry, the dendrimer SPL2923 (BRI2923), which comprises an ammonia core, four generations of PAMAM branches and 24 DNAA surface groups, inhibits not only HIV-1 entry but also recombinant HIV-1 reverse transcriptase (RT) and reverse transcription in HIV-1 infected cells [11]. Accordingly, we investigated whether SPL7013 and SPL7115 inhibit recombinant HIV-1 RT activity (Figure 4A). The SPL7013 50% inhibitory concentration (IC_50_ ± standard error) (0.69±0.04 µg/ml) was similar to SPL2923 (0.48±0.05 µg/ml) while SPL7115 was less active (17.9±0.04 µg/ml). These data demonstrate that lysine-based DNAA dendrimers can also inhibit recombinant HIV-1 RT.

To determine whether SPL7013 and SPL7115 inhibit HIV-1 reverse transcription in HIV-1 infected cells we performed a time of addition assay in the TZM-bl indicator cell line (Figure 4B). The experiments included the controls AMD3100, a CXCR4 antagonist that blocks HIV-1 entry, and nevirapine, a nonnucleoside reverse transcriptase inhibitor (NNRTI). As expected, the addition of nevirapine could be delayed for up to 6 h post infection before loss of its HIV-1 inhibitory activity while AMD3100 failed to block HIV-1 replication if the drug was added after virus addition (Figure 4B). Both concentrations of SPL7013 and SPL7115 behaved similarly to AMD3100 indicating that they inhibit viral entry but not reverse transcription in TZM-bl cells, presumably because they either fail to enter cells or they cannot access RT within the reverse transcription complex. These data demonstrate that both SPL7013 and SPL7115 inhibit HIV-1 entry.

### SPL7013 and SPL7115 block R5 and X4 envelope mediated cell-to-cell fusion

We examined the relative ability of dendrimers to inhibit X4 versus R5 envelope mediated cell-to-cell fusion to gain insights into dendrimer properties required for potent inhibition of cell-associated HIV-1 transmission. SPL7013 inhibited both X4 and R5 envelope mediated fusion with 50% fusion concentrations (FC_50_ ± standard error) of 0.08±0.02 µM (n = 3) and 0.94±0.19 µM (n = 4), respectively (Figure 4C). In contrast, SPL7115 was 4.4-fold (FC_50_ = 0.35±0.07 µM, n = 3, p = 0.1) and >20-fold (FC_50_>18.9 µM, n = 4) less potent than SPL7013 in inhibiting X4 and R5 envelope mediated fusion, respectively (Figure 4D). The fusion inhibitor T-20 (enfurvitide) was included as a positive control and inhibited both X4 and R5 mediated fusion with FC_50_ values of 2.0±0.3 nM and 4±1 nM, respectively while the NNRTI, efavirenz did not inhibit cell-to-cell fusion (Figure S3). These data demonstrate that dendrimers block HIV envelope mediated cell-to-cell fusion. Notably, the G4 dendrimer is more potent than the smaller G2 dendrimer in inhibiting R5 envelope-mediated fusion.

### Regions of gp120 suitable for interactions with dendrimers

**Figure 5:**
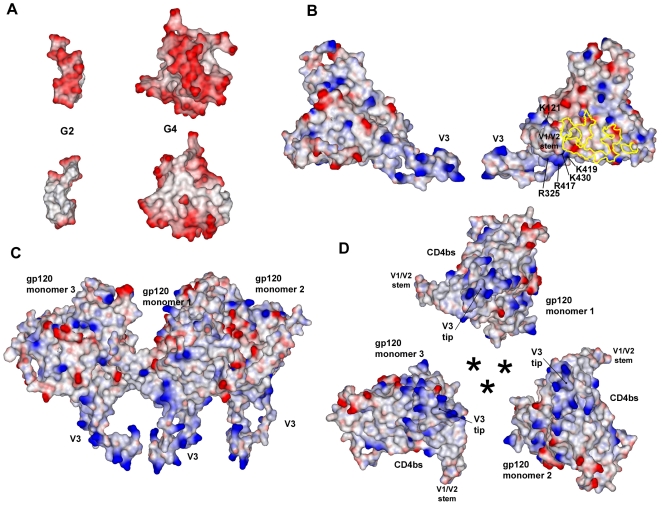
Electrostatic surface views of dendrimer and gp120 models. (**A**) Models of the G2 (SPL7115) and G4 (SPL7013) dendrimers taken from the last frame of a molecular dynamics simulation [31]. Two views are shown (top and bottom) rotated by 180° around the long axis of the dendrimer. (**B**) Two views (rotated by 180°) of the homology model of the DRNL wild-type gp120. CD4 binding site is denoted by a yellow outline and conserved residues K121, R325, R417, K419 and K430 in the CD4i domain are indicated. (**C**) Side-view of the DRNL gp120 trimer with the V3 loops pointing towards the target cell membrane. The space between the gp120 monomers would normally be occupied by gp41. (**D**) End-on view of the DRNL gp120 trimer with the V3 loops facing the viewer. Predicted position of gp41 monomers are marked by an asterisk. In all views, the solvent-accessible surfaces are mapped with regions of negative (red) and positive (blue) electrostatic potential (charge).

Our studies demonstrate that SPL7013 and SPL7115 block HIV-1 entry indicating that they bind to HIV-1 surface proteins and/or host cell receptors required for entry. In this regard, previous studies have demonstrated that the anionic polymer, PRO 2000, harboring naphthalene monosulfonic acid residues, binds to HIV gp120 in addition to CD4 and the CXCR4 chemokine receptor [28], [29]. Moreover, multiple mutations in HIV-1 gp120 are required to develop resistance to dendrimers as observed for the related DNAA dendrimer SPL2923 [11], [30]. Taken together, these studies indicate that HIV-1 gp120 is one of the targets of DNAA based dendrimers. To understand how SPL7115 and SPL7013 bind to gp120 we generated electrostatic surface views of the dendrimer and the gp120 target as either a monomer or a trimer (Figure 5). As previously described [31], L-lysine dendrimers with DNAA caps are predicted to transition from an elongated structure (G2, SPL7115) to a more compact or globular structure in later generations (G4, SPL7013). The DNAA moieties on the dendrimers are predicted to cluster, especially in earlier generations, resulting in a high density of negative charge on one face of the G2 and G4 molecules (Figure 5A).

Electrostatic surfaces of the DRNL gp120 monomer (Figure 5B) and trimer (Figure 5C and D) models were used to examine possible regions of SPL7115 and SPL7013 binding. In general, the two faces of the gp120 monomer displayed an even distribution of positive and negative surface charge suggesting that a polyanionic dendrimer could interact with a variety of regions of broadly distributed positive charge (Figure 5B). In contrast, the V3 loops and regions surrounding the base of the V3 loops were more densely populated with positively charged residues, the former being typical of X4 strains of HIV-1. Since the V3 loops vary in positive charge between X4 and R5 HIV-1 strains, it is likely that the broad potency observed for SPL7013 and SPL7115 is due in part to interactions with conserved clusters of positive charge on gp120. Thus a putative dendrimer binding site on gp120 was identified that comprises basic residues K121, R325, R417, K419 and K430 that are highly conserved in both X4 and R5 strains (Figure S4) and are located at the base of the V3 region in the CD4-induced (CD4i) domain formed by a conformational change following binding of gp120 to CD4 [32]. The V3 loop in concert with the CD4i domain form the chemokine coreceptor binding sites on gp120 [15]. The role of these basic amino acid residues in dendrimer binding needs to be confirmed by mutagenesis studies. Comparisons of the relative sizes and surface groups of the dendrimers (Figure 5A) combined with the gp120 monomers (Figure 5B) and trimers (Figure 5C and D) highlight that it is possible to coat the envelope protein with several of the negatively charged dendrimers (SPL7115 or SPL7013), thereby blocking the ability of the HIV-1 envelope to fuse with cells.

## Discussion

We have shown that dendrimers can be used as a platform to engineer a diverse array of potent HIV and HSV antivirals, and have potential for the development as inhibitors of other viral pathogens. Based on rational design principles and understanding structure-activity relationships, optimized antiviral dendrimers were identified. One of these dendrimers, SPL7013, demonstrated the most potent activity against a broad-spectrum of HIV isolates, HSV-1 and HSV-2 and is the active pharmaceutical ingredient in the formulated microbicide, VivaGel®. Mechanism of action studies demonstrate that SPL7013 blocks entry of both X4 and R5 HIV-1 strains with equal potency and inhibits X4 and R5 envelope mediated cell-to-cell fusion. Modeling studies indicate the potential for electrostatic interactions of dendrimers with gp120. The favorable *in vivo* anti-HSV-2 activity and lack of toxicity demonstrated in this study along with previously reported anti-HIV_Ba-L_ activity in *ex vivo* human cervical and colorectal explant cultures [33], [34], animal safety and efficacy studies [24], [35], and human safety data [36], [37] make VivaGel® a promising microbicide candidate.

Extensive SAR studies were performed to evaluate the optimum dendrimer size and surface group for HIV-1 inhibitory activity. The most potent activity against a X4 strain of HIV-1 was achieved with a dendrimer comprising two generations and capped with the surface groups, DNAA or 3,5-Ph-(SO_3_Na)_2_, imparting the greatest anionic surface charge and hydrophobicity of all of the evaluated surface groups. Notably, the anti-HIV activities of DNAA and 3,5-Ph-(SO_3_Na)_2_ capped dendrimers did not appreciably increase beyond G2 against the NL4.3 strain of HIV-1. Furthermore, the G4 DNAA dendrimer was only 4-fold more potent against the R5 HIV_Ba-L_ strain compared to the G2 DNAA dendrimer. In contrast, SAR studies for anti-HSV-2 activity revealed a different pattern for DNAA-capped dendrimers where potency dramatically increased with size as exemplified by a 140-fold increase in potency of the G4 compared to the G2 DNAA dendrimer. The same pattern with regard to DNAA dendrimer size and potency was also observed for HSV-1 inhibition. These data suggest that dendrimer anionic surface charge in addition to size is important for HSV inhibition. The larger dendrimers could lead to steric hindrance between viral and host cell receptor interactions and play a greater role in HSV compared to HIV inhibition. Taken together, these studies demonstrate for the first time that dendrimers can be engineered for optimal inhibition of HIV-1, HSV-2 and potentially other viral pathogens.

The differences in SAR for HIV-1 and HSV-2 are likely a consequence of the various targets involved in viral entry. Productive HIV entry is dependent on gp120 binding to CD4 and chemokine receptors [15], [38]. HSV entry relies on viral surface glycoproteins gB, gC, gD, gH/gL for attachment and fusion with productive HSV entry dependent on gB and gC binding to negatively charged heparin sulfate present on cell surface proteoglycans [39]. This binding promotes subsequent interaction of gD to one of three classes of surface receptors including herpes virus entry mediator (HVEM, a member of the TNF receptor family), nectin 1 and nectin 2 (members of the immunoglobulin superfamily) or a modified form of heparin sulfate. Interaction of gD with its receptors triggers fusion of the viral and cell lipid membranes which also requires the involvement of gB and the gH/gL heterodimer [39]. SPL7013 blocks HSV adsorption to cells [40] and it is possible that this dendrimer interferes with the initial binding of HSV to heparan sulfate, although this needs to be confirmed. Thus the ability of dendrimers to interfere with electrostatic interactions between viral surface proteins and host cell receptors represents a common mechanism for inhibition of viral entry, where the potency for a particular virus can be increased by the choice of surface group and dendrimer size.

Our studies demonstrate that both SPL7115 and SPL7013 are HIV entry inhibitors with broad-spectrum anti-HIV activity against a wide range of X4 and R5 clinical isolates, different HIV-1 clades, early R5 clinical isolates, HIV-2 and a HIV-1 strain resistant to reverse transcriptase and protease inhibitors. The ability of SPL7013 to inhibit early R5 strains isolated from PBMCs of individuals with an asymptomatic HIV-1 infection (NB25) and an acute seroconverter (NB27) indicate that SPL7013 is likely to be active against transmitted/founder strains of HIV-1 that, similar to early R5 isolates, tend to be more sensitive to inhibition by fusion inhibitors and chemokine antagonists compared to HIV-1 from chronically infected individuals [17], [41].

Notably, we found that SPL7013 was equally potent in the inhibition of both X4 and R5 strains of HIV-1. This is in contrast to what has been described for several linear polyanion-based microbicide candidates that tend to be more potent inhibitors of X4 [18], [19]. Moreover, a previous study is consistent with our more extensive findings demonstrating that SPL7013 has similar potency against HIV-1_IIIB_ (X4 strain) and HIV_Ba-L_ (R5 strain) at both the 50% and 90% inhibitory concentrations [42]. In contrast, the same study demonstrated that PRO 2000 is more potent against HIV-1_IIIB_ versus HIV_Ba-L_, particularly at the 90% inhibitory concentrations [42]. The greater ability of linear polyanions to inhibit X4 versus R5 strains has been attributed to their interaction with the highly positively charged V3 loop on gp120 [18], [19], [42]. It is likely that SPL7013 also interacts with the V3 loop of X4 strains. However, its ability to equally block R5 strains in cell culture assays suggests either an interaction with conserved basic residues in gp120 of both X4 and R5 strains and/or an ability to bind to host cell receptors required for HIV entry. In this regard, PRO 2000 binds to gp120, CD4 and chemokine receptors [28], [29], [43].

Our evaluation of the anti-HIV-1 activity of SPL7013 in the presence of high concentrations of human serum and in the presence of human CVS demonstrate little impact on the ability of SPL7013 to inhibit HIV-1 in cell culture assays. A previous study has demonstrated that SPL7013 is active at pH∼4.0 (present in the female genital tract) and that 1 mg/ml of SPL7013 (representing 1/30 SPL7013 levels in VivaGel®) completely inhibits HIV-1 infection in both viral entry and cell-associated transmission assays in the presence of human seminal plasma [42]. Furthermore we have demonstrated that VivaGel® recovered after vaginal dosing in healthy women retained potent antiviral activity against HIV-1 and HSV-2 in *ex vivo* assays up to 3 h post-dose in all participants, where the average SPL7013 recovery was 1/20 of the original dose. In addition, potent anti-HIV-1 and HSV-2 activity in recovered gel samples up to 3 h post-dose was also observed in assays performed in the presence of seminal plasma [44]. Taken together, these findings suggest that SPL7013 is likely to retain HIV-1 and HSV-2 inhibitory activity in the presence of CVS and semen during coitus.

Mechanism of action studies demonstrate that while G2 and G4 DNAA dendrimers inhibit recombinant HIV-1 reverse transcriptase, they inhibit HIV-1 entry in cell culture assays. In contrast, a previous study has shown that SPL2923, a dendrimer with a different core, PAMAM branches and DNAA surface groups blocks intracellular HIV-1 reverse transcription in addition to viral entry [11]. SPL2923 is predicted to have a different shape and overall surface charge compared to SPL7115 and SPL7013, which may allow it to enter cells in order to inhibit reverse transcription. However, differences in the ability of dendrimers to enter TZM-bl cells used in our study and MT-4 cells used in the study by Witvrouw and colleagues [11] cannot be excluded. Regardless, the ability of dendrimers to block HIV entry is likely to represent their major mechanism of action since their activity for blocking entry is far more potent than concentrations required to inhibit reverse transcription in cells [11]. Moreover, an HIV-1 strain with decreased susceptibility to SPL2923 has been reported that harbors mutations in gp120 but not in the reverse transcriptase indicating lack of selective pressure of the dendrimer against the reverse transcriptase target [11].

Semen harbors both cell-free HIV and HIV infected leukocytes [45]. However, it is unclear whether the sexual transmission of HIV is due to cell-free or cell-associated virus or both, although a recent study suggests the former in men who have sex with men [46]. To examine the potential of dendrimers to block cell-to-cell fusion we evaluated the relative capacity of SPL7115 and SPL7013 to inhibit envelope mediated cell-to-cell fusion. Our studies demonstrate that G2 and G4 DNAA dendrimers block envelope mediated cell-to-cell fusion, although SPL7013 was >20-fold more potent in blocking R5 envelope mediated fusion compared to SPL7115. These data indicate that dendrimer size is more important in blocking R5 compared to X4 envelope mediated fusion. Notably, the CCR5 and CXCR4 antagonists maraviroc and AMD 3100, respectively failed to inhibit fusion in this assay (data not shown) suggesting that the multivalency of dendrimers may be important for inhibiting cell-to-cell fusion. We also observed that SPL7013 was 12-fold more potent in blocking R5 compared to X4 envelope mediated fusion. A more modest difference (∼4-fold) was reported for the ability of SPL7013 to block R5 envelope mediated fusion and R5-tropic cell-associated HIV-1 transmission compared to assays with X4 envelope and virus [42]. The different cell types, constructs and expression levels of CD4, CXCR4 and CCR5 could have accounted for the relative differences between inhibition of X4 versus R5 envelope mediated fusion observed in our studies. This is supported by a report demonstrating that the ability of CCR5 ligands to block HIV infection is dependent on CCR5 expression levels and other host factors [47]. Taken together, studies by us and others clearly demonstrate that SPL7013 blocks both cell-free and cell-associated transmission with X4 and R5 HIV strains [42].

We have shown that VivaGel® does not cause toxicity as measured by an increase in HSV-2 susceptibility when applied to the vagina of the mouse. These data are consistent with lack of toxicity found in VivaGel® phase I safety studies in men and women [36], [37] and *in vitro* studies demonstrating the lack of disruption of intracellular tight-junctions of polarized epithelial cells by SPL7013 formulated at a higher concentration (5% in Carpobol-based aqueous gel) compared to VivaGel® used in this study [27]. Disruption of tight junctions and thus the integrity of the epithelial cell layer in the genital tract by cellulose sulfate (Ushercell) [48] has been proposed as one of the factors that may have contributed to a trend towards increased HIV-1 acquisition in women using cellulose sulfate as a topical microbicide in a randomized, double-blind placebo controlled phase III trial [20]. It has also been proposed that enhancement of HIV-1 infection by cellulose sulfate at threshold antiviral concentrations may have accounted for an increase in HIV-1 acquisition *in vivo* [49]. However, enhancement by cellulose sulfate was not observed using an *ex vivo* model for HIV-1 infection comprising human vaginal epithelial sheets containing CD4+ T lymphocytes and Langherans cells [50]. In addition, studies in PHA stimulated human PBMCs attributed the marginal enhancement of HIV-1 replication observed by SPL7013 and PRO 2000 to an assay artifact and therefore is unlikely to be relevant *in vivo* [51]. This notion has been confirmed in two recent efficacy trials, which did not demonstrate increased HIV acquisition in women using PRO 2000 [22], [52].

Several candidate microbicides from the nonspecific and moderately specific classes have been evaluated in clinical trials and either demonstrate no efficacy or promote HIV acquisition in women [20], [21], [52], [53]. More promisingly, the macromolecular anionic polymer PRO 2000 formulated as a 0.5% gel reduced HIV infections by 30% compared to women using placebo gel or no treatment. While this did not reach statistical significance (*P* = 0.1), an analysis of women who did not use condoms in this same study demonstrated that the product provided 80% efficacy [52]. In marked contrast to the results of this phase IIb PRO 2000 study, a larger phase III trial demonstrated that 0.5% PRO 2000 lacked efficacy in preventing HIV acquisition [22]. Despite the lack of efficacy demonstrated by linear polyanion based microbicide candidates in preventing the sexual transmission of HIV in clinical trials, a phase IIb study of a 1% tenofovir gel demonstrated a statistically significant 39% reduction in HIV infection, proving the concept of a topical gel-based microbicide for HIV prevention [54], but leaving room for significant improvement in effect.

The dendrimer, SPL7013, has a chemically defined structure and a distinct surface group compared to linear polyanion-based microbicides. SPL7013 also has several advantages over the ineffective linear polyanions, including an ability to inhibit R5 virus and lack of effect on cellular tight junctions [27]. SPL7013 is formulated in VivaGel® so that there is ∼20,000-fold excess of dendrimer delivered to the female genital tract compared with the in vitro EC_50_ for HIV clinical isolates and ∼45,000-fold excess of dendrimer compared with the in vitro EC_50_ for a HSV-2 clinical isolate. Compared to 2 g of 0.5% PRO 2000 [55], VivaGel® (3% w/w SPL7013 in 3.5 g) delivers 10.5-fold higher levels of active drug at concentrations that are tolerated in vivo [36]. In contrast to tenofovir and other similar antiretrovirals, SPL7013 demonstrates no systemic absorption (and potential toxicity) [9], and propagation of HIV in cell culture in the presence of SPL7013 for 43 passages results in virus with only a 3-fold decrease in SPL7013 susceptibility compared to wild-type (G. Tachedjian, unpublished data). In addition, antiretrovirals such as tenofovir need to enter target cells present in the sub-epithelial layers, thus leaving the lumen of the female genital tract, where the infection process commences, potentially unprotected. Finally, a product like VivaGel®, if effective, would have the advantage of being more widely available than an antiretroviral drug, which would need a prescription. Therefore, the continued development of a possible over-the-counter microbicide is important.

The data presented in the current study provide a clear rationale and additional supporting data for the development of the dendrimer SPL7013 and VivaGel® as a broad-spectrum microbicide with activity against HIV and HSV, and potentially other sexually transmitted viruses. A future approach that involves combining a microbicide such as VivaGel® (SPL7013) that protects the vaginal lumen and has dual action HIV and HSV inhibitory activity with a specific antiretroviral agent such as tenofovir, which needs to be delivered to target cells in the sub-epithelial layers, could potentially provide greater protection against HIV acquisition than a microbicide with a single active pharmaceutical ingredient.

## Materials and Methods

### Ethics Statement

All experimental protocols in mice were performed in accordance with the standards established by the US Animal Welfare Acts, set forth in NIH guidelines and the Policy and Procedures Manual of Johns Hopkins University Animal Care and Use Committee. The study protocol MO08M334 “Evaluating vaginal microbicides in mice”, which is specific for the mouse experiments described in this study, was approved by the Johns Hopkins University Animal Care and Use Committee (i.e. the Johns Hopkins Animal Ethics Committee) Chaired by Nancy A. Ator. The Johns Hopkins University Animal Assurance number is 3272-01.

### Dendrimer synthesis and purification

#### Dendrimer backbone

The preparation of lysine dendrimers from the benzhydryl amide protected lysine core and the subsequent capping reactions have been described previously [12], [13]. The materials were prepared with a high degree of purity using iterative cycles of Boc deprotection and reaction of the trifluoroacetic (TFA) salts with excess of the *p*-nitrophenol active ester of α,ε-*^t-^*Boc_2_-(L)-lysine. The Boc-protected materials and the multi-TFA salts are both solids that were further purified through washing and precipitation steps. All Boc-protected lysine dendrimers used here (G1–G5) were chemically and physically stable, and were prepared for subsequent surface functionalisation as desired.

#### Surface capping groups

Anionic capping groups were purchased or synthesized as required. 4-Sulfobenzoic acid sodium salt (“4-Ph-SO_3_Na”) was purchased (Sigma-Aldrich), di-*t*-butyl-[[(4-nitrophenyl)oxy]carbonyl]-(L)-glutamate (“glutamic”) was modified as described previously [56], 1,3,5-benzenetricarboxylic acid [“3,5-Ph-(CO_2_Na)_2_”] was purchased (Sigma-Aldrich), 3-sulfo-2-thiophenecarboxylic acid (“2-thio-3-SO_3_Na”) was modified as described previously [57], 3,5-disulfobenzoic acid disodium salt [“3,5-Ph-(SO_3_Na)_2_”] was purchased (Sigma-Aldrich), 2-[(3,6-disulfo-1-naphthalenyl)oxy]-acetic acid disodium salt (“DNAA”) and 2-[(3-sulfo-1-naphthalenyl)oxy]-acetic acid (“MNAA”) were both modified as described previously [58].

### Virus Strains

NL4.3, a X4 strain [59], was derived by transfection of 293T cells with pDRNL using the calcium phosphate method [60]. Virus was propagated at least three times in MT-2 cells prior to use in anti-HIV assays. HIV_Ba-L_ is a R5 laboratory strain, which was propagated in human PBMCs and macrophages. HIV-1 strains 92RW016 (clade A), 92BR025 (clade C), 92UG046 (clade D), CMU02 (clade EA), 93BR020 (clade F), BCF01 (clade O) and HIV-2 were obtained from the NIH AIDS Research and Reference Reagent Program. HIV-1 clinical isolates CB1-br, MACS1-spln, MACS3-LN and MAC3-br were isolated from HIV-1 infected individuals [61] and provided by Dana Gabuzda (Dana-Farber Cancer Institute). Early circulating (NB25 and NB27) and late emerging (NB2 and NB6) R5 isolates [17] were provided by Anthony Cunningham (Westmead Millenium Institute, Sydney, Australia). Apart from NL4.3 and HIV_Ba-L_ all strains were propagated in PHA-stimulated human PBMCs. HSV-1 strain 250735 and HSV-2 strain 250733 are highly cytopathic strains isolated from Australian patients that were typed by the Victorian Infectious Diseases References Laboratory (VIDRL, Melbourne, Australia) and were provided by Dr Chris Birch [14]. HSV-1 (F) and HSV-2 (G) were obtained from the American Type Culture Collection.

### Cells

MT-2 cells [62] were cultured in RPMI1640 and 10% foetal calf serum (FCS) buffered with 25 mM HEPES as previously described [60]. The TZM-bl indicator cell line [63], obtained through the NIH AIDS Research and Reference Reagent Program, and 293T cells were cultured in Dulbecco modified Eagle medium (DMEM) supplemented with 10% FCS, 100 U/ml penicillin, 100 µg/ml streptomycin and 29.2 mM glutamine (DMEM-10). Human embryonic lung (HEL) fibroblasts cells were obtained from VIDRL and were propagated in DMEM-10. PHA-stimulated human PBMCs from uninfected donors were prepared as described previously [64]. Cf2-Luc cells [65], derived from the Cf2th canine thymocyte cell line, stably express the luciferase gene under the control of the HIV-1 promoter and were cultured in DMEM-10 supplemented with 0.7 mg/ml of G418.

### HIV inhibition assays

Initial screening assays to determine the inhibitory activity of dendrimers against NL4.3 were performed in the MT-2 T-lymphocyte cell line using cell viability as the readout as previously described [66] except that cell viability was determined using the CellTitre 96 Aqueous One Solution Reagent (Promega) according to manufacturer's instructions. Each assay included SPL7013 as a positive control for HIV-1 inhibition with assays being deemed valid if the SPL7013 EC_50_ was within two standard deviations of the average value of 0.16 µM. The cytotoxicity of dendrimers was evaluated in parallel, with cultures incubated in the absence of virus. The EC_50_ and CC_50_ values were calculated as published [66].

To evaluate the inhibitory activity of the most potent dendrimers against different HIV-1 clades and clinical isolates, assays were performed in the TZM-bl indicator cell line that expresses CD4 and the CCR5 and CXCR4 chemokine receptors and engineered with the luciferase and β-galactosidase genes under the control of the HIV-1 promoter [63]. Assays were performed as published [60] except that luciferase activity was used to determine HIV replication, which was measured using the Steady Glo Luciferase Assay System (Promega) according to manufacturer's instructions. EC_50_ values were calculated as published [60]. Luminescence was measured using a FLUOStar Optima microplate reader (BMG LABTECH, GmbH, Germany).

SPL7013 inhibition of HIV-1 in human peripheral blood mononuclear cells and in the presence of human serum and cervicovaginal secretions in TZM-bl cells were performed as described in Supplementary Methods S1.

### HSV inhibition assays

The anti-HSV-1 and -HSV-2 activities of dendrimers were determined in HEL cells using cell viability as the readout for viral replication. HEL cells were seeded at 6,000 cells per well of a 96 well plate in DMEM-10 and incubated overnight at 37°C in 5% CO_2_. Following incubation, medium was removed and cells replenished with DMEM supplemented with 2% FCS (DMEM-2) containing dendrimer at twice the final concentration and infected with 500 TCID_50_ of HSV. Cells were incubated for 4–6 days at 37°C in 5% CO_2_ until cytopathic effects were observed in 100% of the cells in cultures containing virus without drug. After incubation, the medium was removed and cells were replenished with 100 µl of DMEM containing 2% FCS after which the cell viability was determined using the CellTiter 96 Aqueous One Solution Reagent (Promega) according to manufacturer's instructions. The dendrimer EC_50_ values were calculated from three independent assays as described previously [66]. The EC_50_ value obtained for SPL7013 in our assay was similar to the value obtained using a plaque reduction assay for HSV [24].

### Mouse model for evaluating protection and susceptibility to HSV-2

The methods for both the protection test and susceptibility test for toxic effects were as previously described [25]. Briefly, female CF-1 mice 6–8 weeks old (Harlan, Indianapolis, IN) were injected subcutaneously with 2.5 mg Depo-Provera® (medroxyprogesterone acetate, Pharmacia & Upjohn Company, Kalamazoo, MI), a treatment that synchronizes the mice into a diestrous-like state that exposes living cells on the entire surface of the vagina, greatly increases HSV-2 susceptibility, and makes mice uniformly susceptible to HSV-2. Strain G of HSV-2 (ATCC lot #3405329)(Virotech International, Rockville, MD) was diluted with Bartels Tissue Culture Refeeding Medium (Trinity Biotech, St Louis, MO) and the viral inoculum delivered with a Wiretrol pipet (Drummond Scientific, Broomall, PA) with a fire polished tip to minimize potential injury.

For the HSV-2 protection test, 20 µl of VivaGel® (or PBS) was delivered to the vagina and the 10 µl viral inoculum containing 10 ID_50_ (∼10^4^ TCID_50_) was delivered after a specified time interval. For each time point 10 mice received VivaGel® and 10 controls received PBS.

For the HSV-2 susceptibility test, 20 µl of VivaGel® (or HEC placebo gel) was delivered to the vagina, and then a low dose inoculum with 0.1 ID_50_ was delivered in 10 µl of Bartels medium 12 h later, a time interval at which prior tests indicate the greatest increase in susceptibility is likely to occur. To determine the relative susceptibility of the mice, a dose-response curve was obtained to observe the fraction of mice infected as a function of the viral dose delivered. The dose-response curve was used to determine the effective ID of the low-dose inoculum in the test animals. Relative susceptibility is defined as the ratio of the effective ID the low-dose inoculum delivered to the test mice divided by the ID it delivered to control animals. A total of 40 mice received VivaGel® and 40 mice received HEC placebo.

For both the HSV-2 protection and susceptibility tests, vaginal lavages were obtained 3 days after inoculation and evaluated for viral shedding. Bartels Medium (50 µl) was delivered to the vagina and pipetted in and out 20 times to maximize viral recovery, then diluted into 50 µl Bartels Medium. The vaginal lavage was clarified by centrifugation (6,500 rpm, 5 min) and the supernatant was placed on human newborn foreskin diploid fibroblast target cells (Biowhitaker, Walkersville, MD). Cytopathic effect was scored 48 h later and mice whose lavage cultures displayed cytopathic effects were considered infected.

### Inhibition of recombinant HIV-1 RT

HIV-1 RT was expressed from pRT6H-PROT and purified as previously described [60]. Assays were performed by pre-incubating 25 ng of HIV-1 RT with the test compound in 50 mM Tris-HCl, pH 7.8, 0.1 mg/ml bovine serum albumin (BSA) and 0.01% IGEPAL for 30 min on ice. The reaction was initiated by adding 200 nM of a 35-nucleotide DNA template [(5′-AGAATGGAAAATCTCTAGCAGTGGCGCCCGAACAG-3′) annealed to a 26-nucleotide DNA primer (5′-CCTGTTCGGGGCCACCTGCTAGAGAT-3′)], 10 µM of a dNTP mixture and 5 µCi of ^33^PdTTP (Perkin Elmer) in 50 mM Tris-HCl, pH 7.8, 60 mM KCl, 2 mM dithiothreitol, 5 mM MgCl_2_, 0.1 mg/ml BSA, 0.01% IGEPAL for 1 h at 37°C. Following incubation, samples were applied to DE81 membrane, washed and incorporated counts quantified using a FLA-2000 phosphorimager (Fujifilm).

### Time of addition assay

TZM-bl cells were seeded in 96-well tissue culture plates at 2.5×10^4^ cells per well in 100 µl of DMEM-10. Cells were grown for 12 h at 37°C in 5% CO_2_ after which triplicate wells were infected with NL4.3 at a multiplicity of infection of one by spinoculation. Briefly, DMEM-10 was removed from cells and replaced with 100 µl of virus in DMEM-10 and plates were centrifuged at 1200 *g* for 60 min at 17°C. Viral inoculum was removed and cells washed twice with 250 µl of cold DMEM-10 after which 100 µl of DMEM-10 or DMEM-10 containing drug was added at t = 0 to appropriate cells. DMEM-10 containing drug was subsequently added at t = 2, 3, 4, 5, 6, 8 and 24 h post infection to appropriate wells. At 31 h postinfection, HIV-1 replication was determined by measuring luciferase activity as above.

### Cell-to-Cell Fusion assays

The gp120 gene from the R5 HIV-1 strain ADA and X4 HIV-1 strain HXB2 was cloned into a HIV-1 Env expression vector. Briefly, the 2.1 kb HIV-1 *env* within *Kpn* I and *Bam* HI restriction sites was cloned from viral genomic DNA into pSVIII-HXB2 Env expression vector [67]. The pcDNA3-CD4, pcDNA3-CCR5 and pcDNA3-CXCR4 plasmids express human CD4, R5 and X4 respectively [61]. pSVTat expresses the HIV-1 Tat protein. Cell-cell fusion assays were conducted as described previously [68]. Briefly, Cf2-Luc target cells seeded in 25 cm^2^ tissue culture flasks were transfected with 1 µg of CD4 plasmid and 3 µg of R5 or X4 plasmid. 293T effector cells seeded in 6-well tissue culture plates were co-transfected with 3.4 µg of ADA or HXB2 gp120-expressing plasmid and 0.6 µg pSVTat. Target and effector cells were transfected using Lipofectamine 2000 (Invitrogen) according to the manufacturers' protocol. Effector cells expressing ADA or HXB2 Env were pre-incubated in DMEM-10 containing 10-fold increasing concentrations of efavarenz, T-20, dendrimer or no drug (untreated controls) for 30 min at 37°C. Approximately 2×10^4^ effector cells were added to 2×10^4^ Cf2-Luc target cells and were incubated at 37°C in replicate wells containing 200 µl of culture. Cells from replicate wells were harvested at 7 h post-mixing and assayed for luciferase activity (Promega) according to the manufacturers' protocol. 293T cells transfected with pSVTat alone were used as negative controls to determine the background level of luciferase activity.

### Generation and computational analysis of dendrimer and gp120 models

Coordinates for the dendrimer models were taken from the last frames of a molecular dynamics simulation [31]. The crystal structure of JRFL gp120 containing the V3 variable loop and bound to CD4 and the X5 Fab antibody fragment was used as the template for CD4-bound model of the DRNL wild-type gp120 protein (Protein Data Bank ID: 2B4C) [69]. Trimeric gp120 homology models were generated using the coordinates of the gp120/CD4/17b crystal structure that had been fitted to a cryo-electron tomography structure of the HIV-1 Env spike (Protein Data Bank ID: 3DNO) [70]. Solvent-accessible surfaces were generated in DS Visualizer version 2.0 (Accelrys, USA) using a probe radius of 1.7 Å. Electrostatic surface potential was calculated by assigning partial charges to all heavy atoms and applying a distance cut-off of 12.5 Å for mapping charges (red for negative and blue for positive) to the molecular surfaces.

### Statistical analysis

The statistical significance of differences between EC_50_ and FC_50_ values obtained from cell culture drug susceptibility and fusion assays were determined using the Wilcoxon rank-sum test. Fisher's exact two-sided test was used to analyze numbers of mice infected and uninfected in test groups vs control groups in the protective efficacy assay.

## Supporting Information

Methods S1 Supplementary Materials and Methods.
(0.05 MB DOC)

Figure S1 Selectivity of L-lysine against the NL4.3 strain of HIV-1 in MT-2 cells. G1–G5 represent generation 1 to generation 5 dendrimers.
(2.66 MB TIF)

Figure S2 HIV-1 inhibitory activity of SPL7013 in the absence and presence of 40% human serum (HS)(A) or different dilutions of human cervical vaginal secretions (CVS)(B). Experiments were performed in the TZM-bl indicator cell line using luciferase as the measure of HIV-1 replication. Fig. S2(A) is representative data from one of two independent assays. Data in Fig. S2(B) were from three independent assays. EC_50_ denotes 50% effective concentration and SE, standard error.
(0.30 MB TIF)

Figure S3 Inhibition of CCR5 and CXCR4 gp120 mediated cell-to-cell fusion by T-20 (enfuvirtide)(A) and efavirenz (B). Data are the average of at least three independent assays. Error bars denote standard error of the mean.
(0.23 MB TIF)

Figure S4 Sequence alignment of gp120 proteins derived from HIV-1 and HIV-2 isolates showing conserved basic residues. The β-2 (A) and β-3 (B) strands of the bridging sheet, V3 loop (C), β-20 (D) and β-21 (E) stands of the bridging sheet are shown. Amino acid sequence is colored according to polarity: Polar, green; Non-polar, black; Positively-charged, blue: Negatively charged, red. Numbered according to NL4.3.
(10.98 MB TIF)

Table S1 Activity of potent dendrimers against the CCR5 strain HIVBaL.
(0.06 MB DOC)

Table S2 SPL7013 has broad-spectrum activity against HIV-1 strains in human PBMC.
(0.07 MB DOC)

Table S3 SPL7013 is active against HIV-2 in PBMCs.
(0.05 MB DOC)

Table S4 SPL7013 is active against a multi-drug resistant HIV-1 strain in PBMCs.
(0.04 MB DOC)
