# Supplementary material for: Structure Activity Relationship of Dendrimer Microbicides with Dual Action Antiviral Activity
Source: PLoS One. 2010 Aug 23;5(8):e12309. doi: 10.1371/journal.pone.0012309 (PMC2925893; doi:10.1371/journal.pone.0012309)
Supplement: Methods S1 — Supplementary Materials and Methods. (0.05 MB DOC) [file pone.0012309.s001.doc]

**Structure Activity Relationship of Dendrimer Microbicides with Dual Action**

**Antiviral Activity**

David Tyssen, Scott A Henderson, Adam Johnson, Jasminka Sterjovski, Katie Moore, Jennifer La, Mark Zanin, Secondo Sonza, Peter Karellas,

Michael P Giannis, Guy Krippner, Steve Wesselingh, Tom McCarthy,

Paul R Gorry, Paul A Ramsland, Richard Cone, Jeremy R A Paull, Gareth R Lewis,

Gilda Tachedjian

**Materials and Methods**

**Inhibitory activity of SPL7013 against HIV strains in human PBMCs**

*Viral isolates and reagents*

Fresh human blood was obtained commercially from Interstate Blood Bank, Inc. (Memphis, TN). The low-passage, lymphotropic clinical isolates HIV-1_TEKI_, HIV-1_SLKA_, HIV-1_ROJO_ and HIV-1_WEJO_ were obtained from pediatric patients attending the AIDS Clinic at the University of Alabama at Birmingham. The clinical isolates ROJO and WEJO were phenotyped as syncytium inducing (SI) in MT-2 cells and the SLKA and TEKI isolates were phenotyped as non-syncytium inducing (NSI) in MT-2 cells. SI and NSI phenotypes have been correlated with lymphocyte and monocyte tropism, respectively, and these viruses have been found to favor the corresponding coreceptor for infection. The multidrug-resistant HIV-1 isolate MDR 769 [1] was obtained from Dr. Thomas C. Merigan (Stanford University). HIV-1 MDR 769 is resistant to the RT inhibitors, zidovudine, didanosine, lamivudine, stavudine, foscarnet and nevirapine and the protease inhibitors indinavir, saquinavir and nelfinavir.

The HIV-1 isolates RW/92/016 (clade A), 302056 (clade B), BR/92/025 (clade C), UG/92/046 (clade D), CMU02 (clade E), BR/93/020 (clade F), JV 1083 (clade G) and BCF01 (clade O) were obtained from the NIH AIDS Research and Reference Reagent Program. The HIV-2 isolates CBL-20 and CDC 310319 were also obtained from the NIAID AIDS Research and Reference Reagent Program. PHA-P was obtained from Sigma (St. Louis, MO) and recombinant IL-2 was obtained from R&D Systems Inc. (Minneapolis, MN).

*Anti-HIV Activity of SPL7013 in PBMCs*

Fresh human PBMCs were isolated from screened donors, seronegative for HIV and hepatitis B virus. Cells were pelleted/washed 2-3 times by low speed centrifugation and resuspended in Dulbecco’s phosphate buffered saline (PBS) to remove contaminating platelets. The leukophoresed blood was then diluted 1:1 with PBS and layered over 14 ml of Ficoll-Hypaque density gradient (Lymphocyte Separation Medium, Cell Grow #85-072-CL, density 1.078 +/- 0.002 gm/ml) in a 50 ml centrifuge tube and then centrifuged for 30 min at 600*g*. Banded PBMCs were gently aspirated from the resulting interface and subsequently washed twice with PBS by low speed centrifugation. After the final wash, cells were counted by trypan blue exclusion and resuspended at 1 x 10^7^ cells/ml in RPMI 1640 supplemented with 15% Fetal Bovine Serum (FBS), 2 mM L-glutamine, 4 µg/ml PHA-P. The cells were allowed to incubate for 48-72 h at 37°C. After incubation, PBMCs were centrifuged and resuspended in RPMI 1640 with 15% FBS, 2 mM L-glutamine, 100 U/ml penicillin, 100 µg/ml streptomycin, 10 µg/ml gentamycin, and 20 U/ml recombinant human IL-2. PBMCs were maintained in this medium at a concentration of 1-2 x 10^6^ cells/ml with biweekly medium changes until used in the assay protocol. Monocytes were depleted from the culture as the result of adherence to the tissue culture flask.

For the standard PBMC assay, PHA-P stimulated cells from at least two normal donors were pooled, diluted in fresh medium to a final concentration of 1 x 10^6^ cells/ml, and plated in the wells of a 96-well round bottom microplate at 50 µl/well (5 x 10^4^ cells/well). SPL7013 dilutions were prepared at a two times the final concentration in microtiter tubes and 100 µl of each concentration was placed in appropriate wells in a standard format. Fifty microlitres of a predetermined dilution of virus stock was placed in each test well (final MOI ≅ 0.1). Wells with cells and virus alone were used for virus control. Separate plates were prepared identically without virus for SPL7013 cytotoxicity studies using an MTS assay system (described below). The PBMC cultures were maintained for seven days following infection, at which time cell-free supernate samples were collected and assayed for reverse transcriptase activity as described below.

*Reverse transcriptase activity assay*

A microtiter plate-based reverse transcriptase (RT) reaction was utilized [2]. Tritiated thymidine triphosphate (^3^H-TTP, 80 Ci/mmol, NEN) was received in 1:1 dH_2_O:ethanol at 1 mCi/ml. PolyrA:oligodT template-primer (Pharmacia) was prepared as a stock solution by combining 150 µl polyrA (20 mg/ml) with 0.5 ml oligodT (20 units/ml) and 5.35 ml sterile dH_2_O followed by aliquoting (1.0 ml) and storage at -20°C. The RT reaction buffer was prepared fresh on a daily basis and consisted of 125 µl 1.0 M EGTA, 125 µl dH_2_O, 125 µl 20% Triton X100, 50 µl 1.0 M Tris (pH 7.4), 50 µl 1.0 M DTT, and 40 µl 1.0 M MgCl_2_. The final reaction mixture was prepared by combining 1 part ^3^H-TTP, 4 parts dH_2_O, 2.5 parts polyrA:oligodT stock and 2.5 parts reaction buffer. Ten microliters of this reaction mixture was placed in a round bottom microtiter plate and 15 µl of virus containing supernatant was added and mixed. The plate was incubated at 37°C for 60 min. Following incubation, the reaction volume was spotted onto DE81 filter-mats (Wallac), washed 5 times for 5 min each in a 5% sodium phosphate buffer or 2X saline sodium citrate (SSC, Life Technologies), 2 times for 1 min each in distilled water, 2 times for 1 min each in 70% ethanol, and then dried. Incorporated radioactivity (counts per minute, CPM) was quantified using standard liquid scintillation techniques.

*MTS staining for PBMC viability to measure cytotoxicity*

At assay termination, the uninfected assay plates were stained with the soluble tetrazolium-based dye MTS (CellTiter 96 Reagent, Promega) to determine cell viability and quantify SPL7013 toxicity as in manufacturer’s protocol. The plate was read spectrophotometrically at 490/650 nm with a Molecular Devices Vmax plate reader

*Validation of Assay Performance*

The overall assay performances were validated by the MOI-sensitive positive control compound, zidovudine (AZT), exhibiting the expected level of antiviral activity (EC_50_ in the range of ≤0.01 µM). Macroscopic observation of the cells in each well of the microtiter plate confirmed the cytotoxicity results obtained following staining of the cells with the MTS metabolic dye.

**HIV inhibitory activity of SPL7013 in the presence of human serum and human cervicovaginal secretions**

Cervicovaginal secretions (CVS) were obtained from eight healthy female donors using the Instead Cup as previously described [3]. Immediately after sampling the Instead cup was placed in a 50 ml centrifuge tube and stored in a closed ice bucket until all samples were obtained. Prior to centrifugation the tube was opened and the dome of the Instead Cup was cut with a sterile scalpel from the dependent bend of the rim up about a third of the way up the dome. The Instead Cup was placed back in the tube and the tube and contents subjected to centrifugation at 600*g* for 15 min at 4^o^C in a swinging bucket table-top centrifuge to retrieve the CVS from the cup. Samples were diluted in OptiMEM serum free medium (containing 50 µg/ml gentamicin, 10 µg/ml ciprofloxacin and 2.5 µg/ml amphotericin B) equal to 10 times the sample’s weight. All diluted samples were then pooled in a 50 ml centrifuge tube and centrifuged at 2000*g* for 15 min at 4^o^C to pellet particulate material. The supernatant was recovered without disturbing the pellet and transferred to a fresh centrifuge tube. Samples were then dispensed as 1 ml aliquots and stored at -80^o^C prior to use in the antiviral assays.

Assays were performed with the NL4.3 strain of HIV-1 in the TZM-bl indicator cell line. TZM-bl cells were seeded at 2 x 10^4^ cells in each well of 96 well culture trays in DMEM-10 and incubated overnight at 37^o^C in 5% CO_2_. Media was aspirated and cells replenished with 20 µl of diluted CVS or AB+ heat inactivated human serum to triplicate wells, 10 µl of medium containing dendrimer at four times the final concentration and 10 µl of NL4.3 (~500 infectious units) in the presence of 40 µg/ml DEAE-Dextran. Cells were incubated for 2 – 3 h at 37^o^C in 5% CO_2_ after which the medium was removed and cells replenished with 200 µl of DMEM-10 and incubated for a further 48 h at 37^o^C in 5% CO_2_. Following incubation cells were lysed and luciferase activity determined using the Steady-Glo Luciferase Assay System (Promega) according to manufacturer’s instructions. Luciferase activity was measured using the FLUOStar Optima Multifunction microplate reader (BMG Labtech). The SPL7013 EC_50_ values were calculated by plotting the log drug concentration versus the percent inhibition of luciferase activity (using Xlfit4 Excel Add-in v.4.2.2 Build 18, mathIQv2.1.2, IDBS, Surrey, UK) compared to the virus control incubated with the corresponding levels of CVS or serum in the absence of dendrimer.

**Supplementary References**

(1) Palmer S, Shafer RW, Merigan TC (1999) Highly drug-resistant HIV-1 clinical isolates are cross-resistant to many antiretroviral compounds in current clinical development. AIDS 13: 661-667.

(2) Buckheit Jr RW, Swanstrom R (1991) Characterization of an HIV-1 isolate displaying an apparent absence of virion-associated reverse transcriptase activity. AIDS Res. and Hum. Retroviruses 7: 295-302 .

(3) Boskey ER, Moench TR, Hees PS, Cone RS (2003) A self-sampling method to obtain large volumes of undiluted cervicovaginal secretions. Sex. Transm. Dis*.* 30: 107-109.
