# Supplementary figures and images for: Structure Activity Relationship of Dendrimer Microbicides with Dual Action Antiviral Activity
Source: PLoS One. 2010 Aug 23;5(8):e12309. doi: 10.1371/journal.pone.0012309 (PMC2925893; doi:10.1371/journal.pone.0012309)

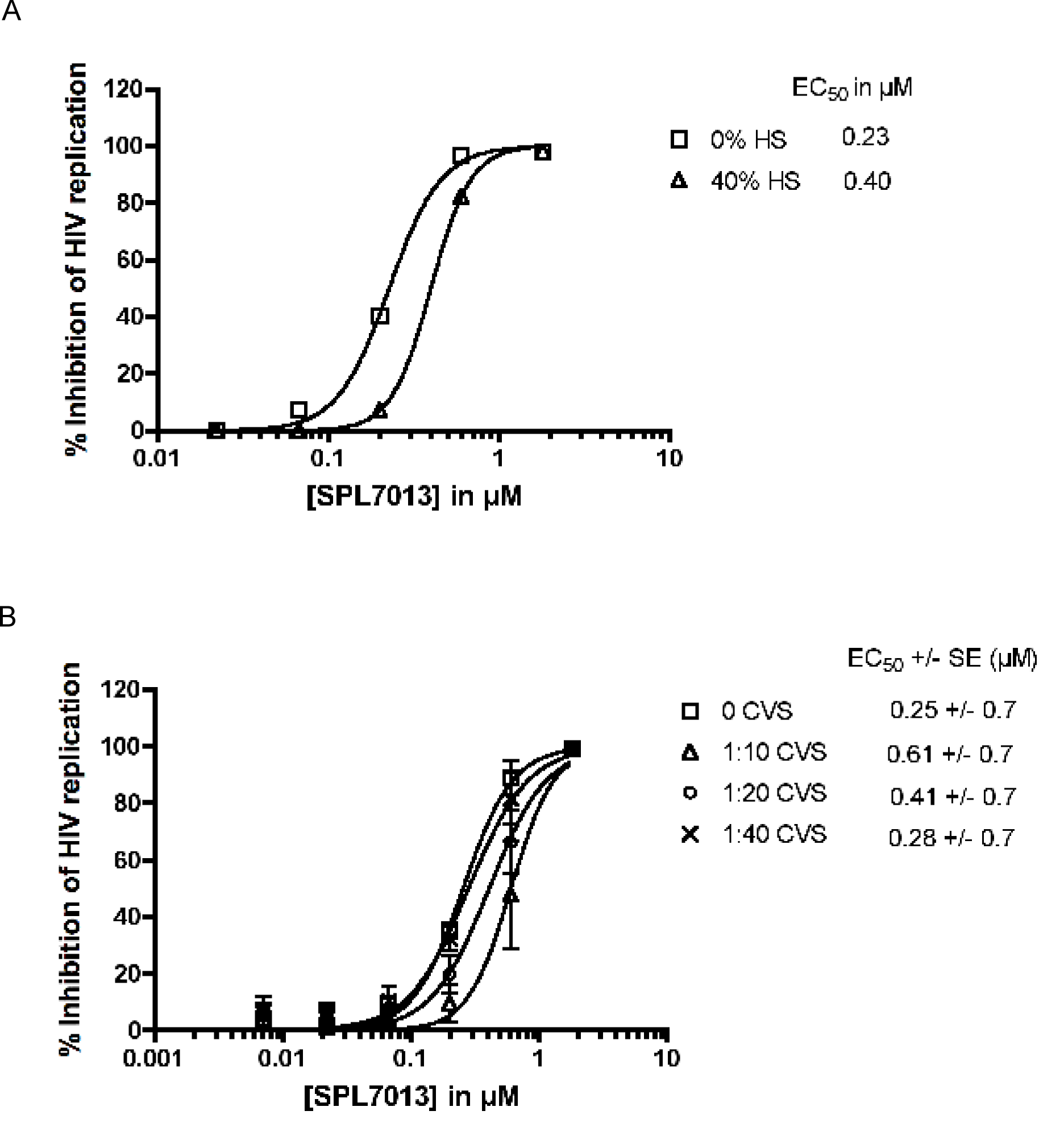

Supplement: Figure S2 — HIV-1 inhibitory activity of SPL7013 in the absence and presence of 40% human serum (HS)(A) or different dilutions of human cervical vaginal secretions (CVS)(B). Experiments were performed in the TZM-bl indicator cell line using luciferase as the measure of HIV-1 replication. Fig. S2(A) is representative data from one of two independent assays. Data in Fig. S2(B) were from three independent assays. EC50 denotes 50% effective concentration and SE, standard error. (0.30 MB TIF) [file pone.0012309.s003.tif]

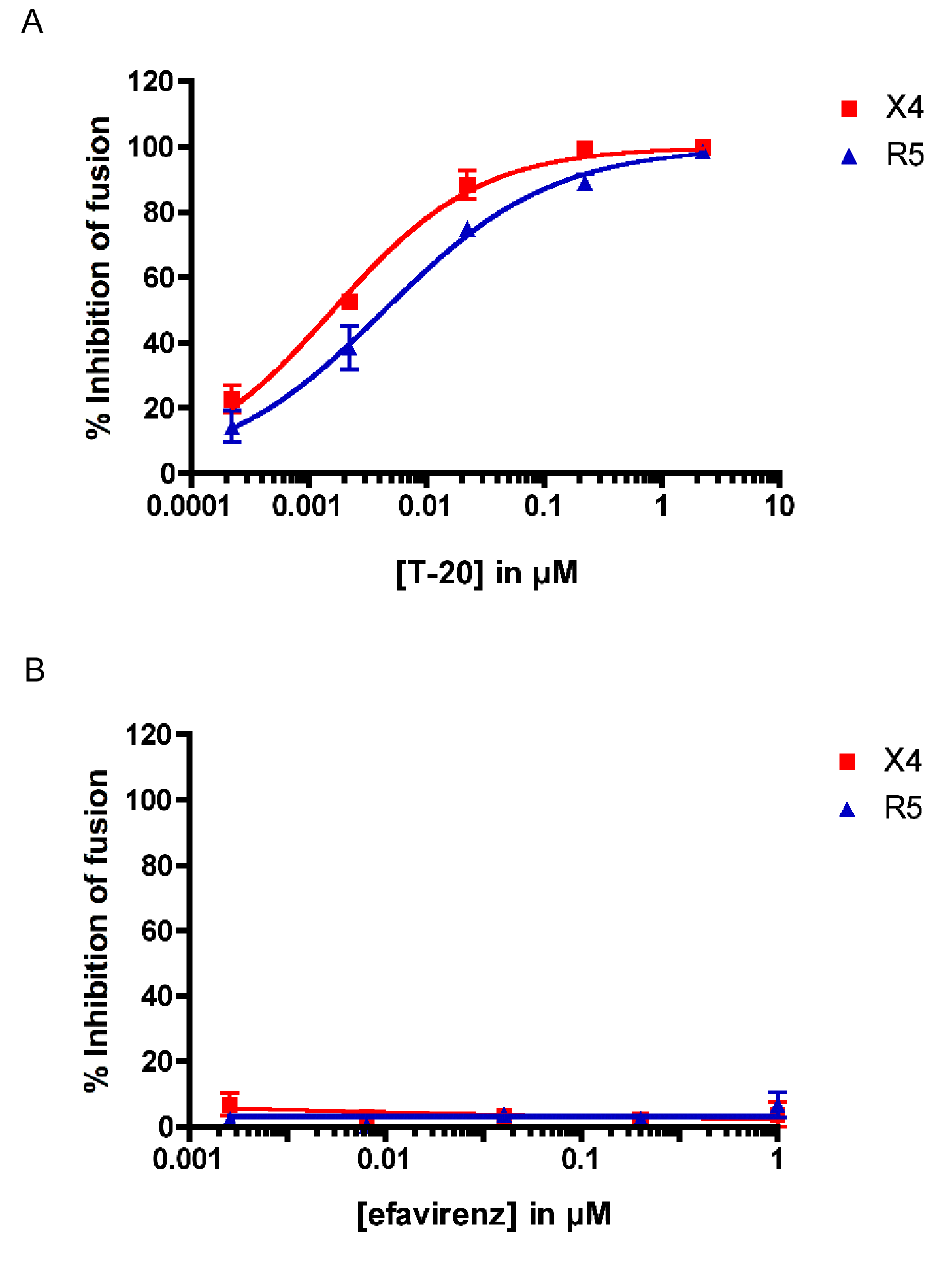

Supplement: Figure S3 — Inhibition of CCR5 and CXCR4 gp120 mediated cell-to-cell fusion by T-20 (enfuvirtide)(A) and efavirenz (B). Data are the average of at least three independent assays. Error bars denote standard error of the mean. (0.23 MB TIF) [file pone.0012309.s004.tif]

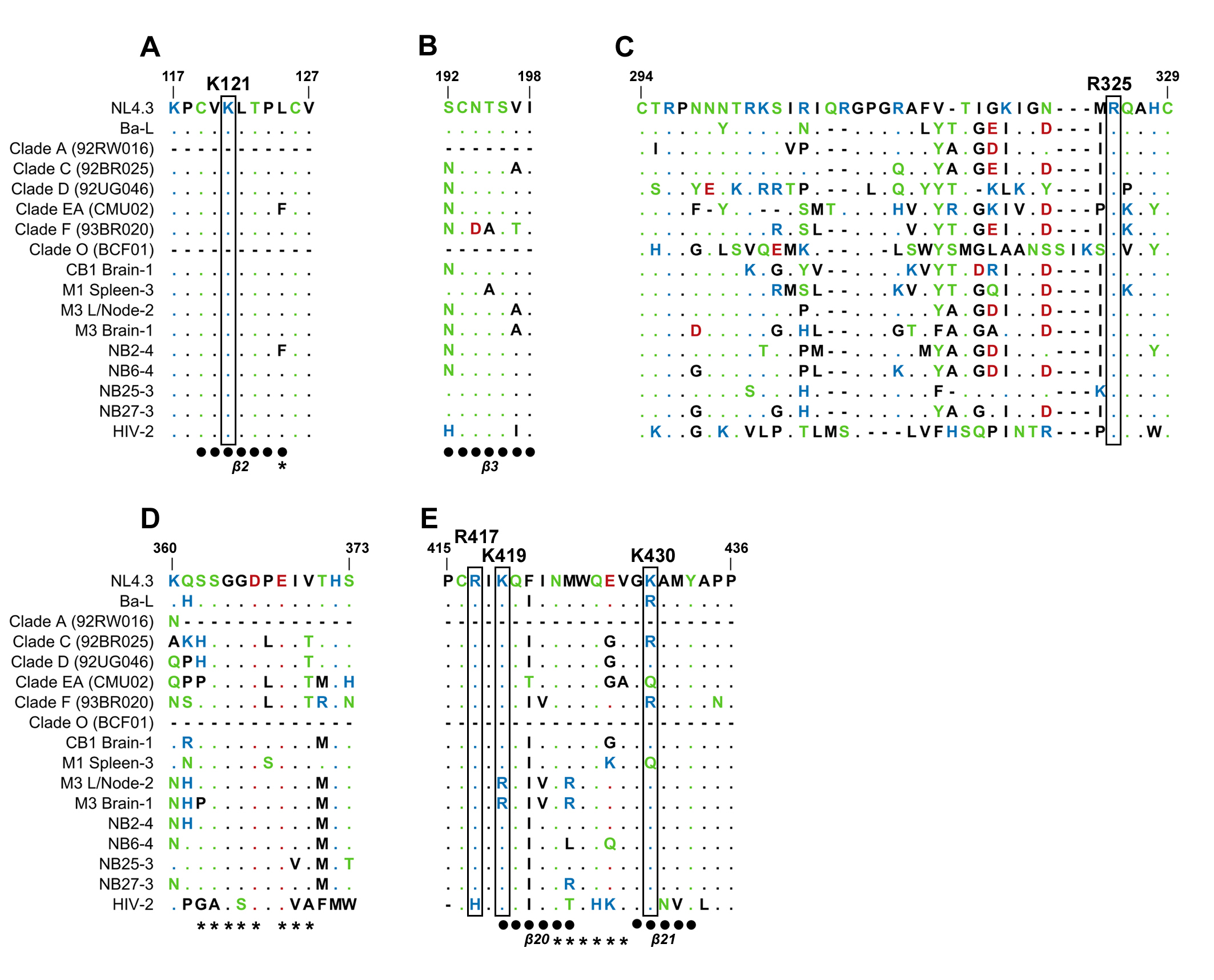

Supplement: Figure S4 — Sequence alignment of gp120 proteins derived from HIV-1 and HIV-2 isolates showing conserved basic residues. The β-2 (A) and β-3 (B) strands of the bridging sheet, V3 loop (C), β-20 (D) and β-21 (E) stands of the bridging sheet are shown. Amino acid sequence is colored according to polarity: Polar, green; Non-polar, black; Positively-charged, blue: Negatively charged, red. Numbered according to NL4.3. (10.98 MB TIF) [file pone.0012309.s005.tif]
