# Supplementary material for: Structure Activity Relationship of Dendrimer Microbicides with Dual Action Antiviral Activity
Source: PLoS One. 2010 Aug 23;5(8):e12309. doi: 10.1371/journal.pone.0012309 (PMC2925893; doi:10.1371/journal.pone.0012309)
Supplement: Table S1 — Activity of potent dendrimers against the CCR5 strain HIVBaL. (0.06 MB DOC) [file pone.0012309.s006.doc]

**Table S1. Activity of potent dendrimers against the CCR5 strain HIV_Ba-L_**

| Dendrimer | Generations | Surface Group | NL4.3 (CXCR4)  EC_50_ ± SE ^a^  (µM) | Ba-L (CCR5)  EC_50_ ± SE ^a^  (µM) |
| --- | --- | --- | --- | --- |
| SPL7115 | 2 | DNAA | 0.18 ± 0.05 | 1.08 ± 0.28 |
| SPL7135 | 3 | DNAA | 0.38 ± 0.10 | 0.64 ± 0.10 |
| SPL7013 | 4 | DNAA | 0.2 ± 0.04 | 0.26 ± 0.05 |
| SPL7615 | 2 | Ph-3,5-(SO_3_Na)_2_ | 0.66 ± 0.14 | 1.71 ± 0.33 |
| SPL7698 | 3 | Ph-3,5-(SO_3_Na)_2_ | 0.31 ± 0.10^b^ | 0.79 ± 0.03 |
| SPL7681 | 3 | Ph-4-SO_3_Na | 0.37 ± 0.13^b^ | 1.42 ± 0.25 |

^a^50% effective concentration (EC_50_) was determined in the TZM-bl indicator cell line from at least three independent assays unless otherwise indicated. SE denotes standard error.

^b^Data obtained from two independent assays.
