# Supplementary material for: Structure Activity Relationship of Dendrimer Microbicides with Dual Action Antiviral Activity
Source: PLoS One. 2010 Aug 23;5(8):e12309. doi: 10.1371/journal.pone.0012309 (PMC2925893; doi:10.1371/journal.pone.0012309)
Supplement: Table S2 — SPL7013 has broad-spectrum activity against HIV-1 strains in human PBMC. (0.07 MB DOC) [file pone.0012309.s007.doc]

**Table S2. SPL7013 has broad-spectrum activity against HIV-1 strains in human PBMC**

| HIV-1 Strain | CXCR4 | CCR5 | Dual  Tropic | SPL7013 (µM)  EC_50_^a^ SI^b^ | | AZT (µM)  EC_50_ SI | |
| --- | --- | --- | --- | --- | --- | --- | --- |
| Clade A (92RW016) |  | + |  | 0.10 | >60 | 0.002 | >500 |
| Clade B (302056) |  | + |  | 0.04 | >137 | 0.01 | >100 |
| Clade C (92BR025) |  | + |  | 0.104 | >58 | 0.004 | >250 |
| Clade D (92UG046) | + |  |  | 0.05 | >676 | 0.003 | >333 |
| Clade EA (CMU02) | + |  |  | 0.09 | >66 | 0.008 | >125 |
| Clade F (93BR020) |  |  | + | 0.08 | >73 | 0.002 | >500 |
| Clade G (JV1083) |  | + |  | 0.15 | >41 | 0.004 | >250 |
| Clade O (BCF01) |  | + |  | 0.02 | >1,348 | 0.004 | >250 |
| ROJO (SI)^c^ | + |  |  | 0.17 | >36 | 0.002 | >500 |
| TEKI (NSI)^d^ |  | + |  | 0.098 | >61 | 0.002 | >500 |
| SLKA (NSI)^d^ |  | + |  | 0.13 | >46 | 0.004 | >250 |
| WEJO (SI)^c^ | + |  |  | 0.15 | >40 | 0.0003 | >3333 |
| Mean EC_50_± SE | 0.12±0.03^e^ | 0.09±0.05^e^ |  |  |  |  |  |

^a^50% effective concentration determined in PBMCs.

^b^Selectivity index (SI) determined by dividing the EC_50_ by the 50% cytotoxic concentration

(CC_50_). CC_50_ is not shown.

^c^Syncytial inducing HIV-1 strain

^d^Nonsyncytial inducing HIV-1 strain.

^e^No significant difference between average EC_50_ values for CXCR4 and CCR5

isolates (*p* = 0.18).
