# Supplementary material for: Structure Activity Relationship of Dendrimer Microbicides with Dual Action Antiviral Activity
Source: PLoS One. 2010 Aug 23;5(8):e12309. doi: 10.1371/journal.pone.0012309 (PMC2925893; doi:10.1371/journal.pone.0012309)
Supplement: Table S3 — SPL7013 is active against HIV-2 in PBMCs. (0.05 MB DOC) [file pone.0012309.s008.doc]

**Table S3. SPL7013 is active against HIV-2 in PBMCs**

| HIV-2 Strain | SPL7013 (µMl)  EC_50_^a^ SI^b^ | | AZT (µM)  EC_50_ SI | |
| --- | --- | --- | --- | --- |
| CBL-20 | 0.29 | >21 | 0.001 | >1000 |
| CDC 310319 | 0.24 | >25 | 0.001 | >1000 |

^a^50% effective concentration determined in PBMCs.

^b^Selectivity index (SI) determined by dividing the EC_50_ by the 50% cytotoxic concentration

(CC_50_). CC_50_ is not shown.
