# Supplementary material for: Structure Activity Relationship of Dendrimer Microbicides with Dual Action Antiviral Activity
Source: PLoS One. 2010 Aug 23;5(8):e12309. doi: 10.1371/journal.pone.0012309 (PMC2925893; doi:10.1371/journal.pone.0012309)
Supplement: Table S4 — SPL7013 is active against a multi-drug resistant HIV-1 strain in PBMCs. (0.04 MB DOC) [file pone.0012309.s009.doc]

**Table S4. SPL7013 is active against a multi-drug resistant HIV-1 strain in PBMCs**

| HIV-1 Strain | SPL7013 (µM)  EC_50_^a^ SI^b^ | | Dextran Sulfate (µg/ml)  EC_50_ SI | |
| --- | --- | --- | --- | --- |
| MDR769 | 0.31 | >19 | 1.25 | >80 |

^a^50% effective concentration determined in PBMCs.

^b^Selectivity index (SI) determined by dividing the EC_50_ by the 50% cytotoxic concentration

(CC_50_). CC_50_ is not shown.
